# Two types of redundancy in multimedia learning: a literature review

**DOI:** 10.3389/fpsyg.2023.1148035

**Published:** 2023-05-04

**Authors:** Melanie Trypke, Ferdinand Stebner, Joachim Wirth

**Affiliations:** ^1^Institute of Educational Science, University of Osnabrück, Osnabrück, Germany; ^2^Institute of Educational Research, Ruhr University Bochum, Bochum, Germany

**Keywords:** redundancy effect, cognitive load theory, cognitive theory of multimedia learning, multimedia instruction, review

## Abstract

Regarding the redundancy effect in multimedia learning environments, more consistency is needed in the theoretical assumptions and investigation of this effect. Current research lacks a comprehensive account of different redundant scenarios in which materials facilitate or inhibit learning and provides little conceptual guidance on how learning processes are affected by different types of redundancy. Theoretical assumptions refer to redundancy as a contentual overlap of information provided by the learning material; in this case, processing duplicated information strains the learners’ limited cognitive capacities. Other assumptions refer to the role of processing limitations in working memory channels, including separate processing for visual and verbal information. In this case, an ineffective combination of sources leads to an overload of the limited working memory capacity. This paper reviews empirical research on the redundancy effect (63 studies) and classifies two types of redundancy: (1) *content redundancy*, and (2) *working memory channel redundancy.* From an instructional psychology perspective, the analyses reveal four different implementations of redundant scenarios: (1) adding narration to visualizations, (2) adding written text to visualizations, (3) adding written text to narration, and (4) adding written text to narrated visualizations. Regarding the effects of the two redundancy types within these scenarios, analyses indicate positive effects of content redundancy (affected by learners’ prior knowledge), negative effects of working memory channel redundancy (regarding visualizations and written text), and positive effects of working memory channel redundancy (regarding narration and written text). Moreover, results point to factors that might moderate the effect of redundancy and illustrate interactions with existing multimedia effects. Overall, this review provides an overview of the state of empirical research and reveals that the consideration of both redundancy types provides further explanations in this field of research.

## 1. Introduction

Research in multimedia learning has investigated multimedia effects and derived principles for designing multimedia learning environments (Mayer, 2014). One of the most prominent effects is the redundancy effect, which appears when different sources of learning material provide redundant information. In investigations of this effect, two theories have played a crucial role: cognitive load theory (CLT; Chandler and Sweller, 1991) and the Cognitive Theory of Multimedia Learning (CTML; Mayer et al., 2001). According to CLT, the redundancy effect occurs when different sources provide the same or unnecessary information (Chandler and Sweller, 1991; Kalyuga et al., 1999). When differentiating between the same and unnecessary information concerning redundancy, the key factor is relevance. The *same* information refers to repeating information related to the learning task. As an example, Chandler and Sweller (1991) pointed to self-explanatory diagrams accompanied by textual instructions that redescribe the content of the diagrams. In this example, the textual instructions do not provide additional information or explanation; they merely repeat the same information. By contrast, *unnecessary* information refers to information unrelated to the learning content for example, by adding background music or entertaining graphics to self-explanatory diagrams (CTML call these “seductive details”). At this point, it is important to consider that CLT suggests that redundant information (both the same and unnecessary) interferes with learning and should be eliminated. From a CTML perspective the elimination of unnecessary information (seductive details) aligns with the coherence principle. According to CTML, the redundancy effect refers “*to any multimedia situation in which learning from animation (or illustration) and narration is superior to learning from the same materials along with printed text that matches the narration*” (Mayer et al., 2001, p. 153).

Both CLT and CTML hold that redundancy impedes learning, though they make different theoretical assumptions (which will be further discussed in section two). Numerous studies support this idea (e.g., Kalyuga et al., 1999; Mayer et al., 2001; Jamet and Bohec, 2007; Austin, 2009). However, many studies report empirical results suggesting that redundancy enhances learning (e.g., Adegoke, 2017) or has no effect on learning (e.g., Chu, 2006). According to Kalyuga and Sweller (2014), there might be two reasons for these contradictory findings. One might be that researchers use the same term—redundancy—to investigate different variations of the effect, such as complementary redundancy^1^ (e.g., Fenesi and Kim, 2014), partial redundancy^2^ (e.g., Roscoe et al., 2015), or concise redundancy^3^ (e.g., Wu, 2011). The other reason might be that some studies implement redundancy between narration and written text (e.g., Diao and Sweller, 2007), others among visualization, narration, and written text (e.g., Mayer and Johnson, 2008), and still others between visualization and written text (e.g., Torcasio and Sweller, 2010). In alignment with Kalyuga et al. (2003), we think that the assumptions of CLT and CTML complement each other and clarify different aspects of redundancy. To consider both perspectives, we suggest distinguishing between two different types of redundancy concerning the content in the learning material (e.g., providing duplicated or unnecessary information) and the included working memory channels to deal with this information.

Therefore, this literature review examines various redundant scenarios to clarify whether differences in content, working memory channels, or both might account for the heterogeneous findings in this research field.

The idea of different types of redundancy has been introduced previously (see Hsia, 1974; Kalyuga et al., 2003; Bohec and Jamet, 2008; Mayer, 2009; Dooley, 2015). However, while earlier classifications emphasized total or partial duplications of spoken and written text (Bohec and Jamet, 2008; Dooley, 2015), the borders of information processing capacity (Miller, 1956; Hsia, 1974), or the logical relationships among different sources of information (Low et al., 2013; Kalyuga, 2014), we propose to look more closely at different redundant scenarios.

To define our two types of redundancy, we focus on cognitive processes localized in the working memory (Schnotz, 2005). Imagine a multimedia environment where learners receive an animation about lightning formation accompanied by a narration that redescribes the content of the animation. A redundancy effect could occur at the cognitive processing level, as cognitive resources are unnecessarily used to determine that the information was duplicated (content redundancy). The same applies to unnecessary information; for instance, learners receive an animation about lightning formation accompanied by background music (not related to the learning content), or learners receive a written text about lightning formation accompanied by entertaining pictures (not related to the learning content). In this case, cognitive resources are unnecessarily used to determine that the information was irrelevant.

The second type of redundancy affects working memory channels. Imagine a situation where learners receive narration and written text. A redundancy effect would affect the processing channel for verbal information in working memory because the narration competes with the written text (Mayer, 2009). Another scenario includes the presentation of visualization and written text; because the two sources are both perceived visually, a redundancy effect could affect the cognitive processing of working memory channels because visualization and written text “compete for limited cognitive resources in the visual channel” (Mayer and Johnson, 2008, p. 124).

Furthermore, it must be considered that these types of redundancy can occur in combination. Imagine a multimedia environment containing visualization and written text. If both sources provide identical information, we have content redundancy and working memory channel redundancy. By contrast, if the visualization and written text contain different information (e.g., the written text complements or supplements information), we have working memory channel redundancy without content redundancy. For instance, learners receive an animation on how to assemble a piece of furniture. The animation would show each step of the assembly process, e.g., how to attach two parts, while the written text would provide more detailed instructions on how much pressure to apply or how to tighten the screws.

Since redundancy must be considered by anyone involved in designing multimedia materials (including instructional designers and educators), this review synthesizes existing research, applying current insights to different variations of the effect on a theoretical level. The distinction of two redundancy types combines the assumptions of CLT and CTML and enables us to categorize the implementations among different studies. This leads to an opportunity to fill gaps in the current understanding and highlight areas that need further research. On a practical level, this categorization enables us to elucidate factors that influence the effectiveness of redundant information and its relation to other principles of multimedia learning, which leads to implications for the design of instructional materials in terms of how to optimize the use of redundant information, for example, when to use which type of redundancy and when a type might be counterproductive.

## 2. Theoretical derivation of the two redundancy types

In the following, we describe the theoretical assumptions that provide the basis for the derivation of our proposed redundancy types. Regarding our classification, the assumptions of CLT (Chandler and Sweller, 1991; Sweller, 2010) align with our concept of “content redundancy” (e.g., the duplication of relevant information).

Cognitive load theory is an instructional theory about cognitive information processing demands on human working memory during learning and problem-solving. Research in this area focuses on the limited capacity of working memory, aiming to identify the most effective design recommendations for learning environments (Sweller et al., 2011). According to CLT, the redundancy effect occurs when two sources provide identical or unnecessary information or when multiple pieces of information that can be understood independently are presented concurrently (e.g., Chandler and Sweller, 1991; Kalyuga et al., 1999; Kalyuga, 2014). Before CLT established this definition of redundancy, prior studies investigated the split-attention effect (e.g., Tarmizi and Sweller, 1988). Both the redundancy effect and the split-attention effect deal with multiple sources of information (e.g., visualization and written text) and the associated increase in extraneous cognitive load (Sweller et al., 2011). However, unlike the redundancy effect, the split-attention effect only occurs if two sources of information are unintelligible in isolation but are each essential to achieving the learning goal (Sweller et al., 2011).^4^ In cases of redundancy, learners must process unnecessary information, since some information is either identical to already-processed information or unnecessary for learning (CTML refers to the exclusion of unnecessary information as the “coherence principle”; Mayer and Fiorella, 2014). Overall, according to CLT, redundancy of any kind is harmful to learning, but the extent of detriment depends on the complexity of the learning material. The complexity of learning material derives from the degree of element interactivity (Sweller, 2010). If elements of the learning material are interrelated, they must be stored and processed simultaneously in working memory. In this case, CLT implies a high level of element interactivity and high working memory requirements for information processing (Sweller, 2010). If redundant information is added to learning material that contains high element interactivity, it is more likely to be detrimental because it adds extraneous cognitive load that may overload working memory capacity. In contrast, when element interactivity is low, additional redundant information may not lead to an overload of working memory capacity.

Our understanding of *working memory channel redundancy* aligns with the assumptions of the CTML (Mayer, 2014) because it focuses on processing information from multiple sources in the same working memory channel. Regarding verbal redundancy, CTML also includes the concept of *content redundancy* when it refers to narration with duplicated written text.

Cognitive theory of multimedia learning is a theory of learning from instructional messages, and it aims to develop instructional design guidelines that enable active processing in learners and promote the construction of meaningful internal representations of learning content. This perspective (Mayer, 2009) uses the term redundancy in a more restricted way and represents a subset of CLT’s broad definition (Mayer et al., 2001). For CTML, the redundancy effect occurs when information is presented via visualization, narration, and written text (Moreno and Mayer, 2002). In such cases, redundancy presumably harms learning because “(a) the visual channel can become overloaded by having to visually scan between pictures and on-screen text, and (b) because learners expend mental effort in trying to compare the incoming streams of printed and spoken text” (Mayer, 2009, p. 118). Mayer et al. (2001) and Moreno and Mayer (2002) identified evidence for this assumption. They indicated that learners perform worse when learning with visualization, narration, and written text than with visualization and narration only. However, Mayer and Johnson (2008) revised their definition of redundancy after demonstrating the benefits of embedding written keywords from a narrated text into visual displays. These benefits were limited to improved performance on a memory test. The most common explanation for these results is that adding written keywords instead of the full written text to the visual part of multimedia instruction can guide learners’ attention toward relevant information (Mayer, 2014).

Further, CTML defines a subclass of redundant information, *verbal redundancy*. Verbal redundancy refers to the presentation of identical words in both written text and narration, with no visualizations (Mayer, 2009). Research on verbal redundancy indicates that the simultaneous presentation of verbal information as written text and narration results in improved learning performance over narration alone, if the written information is short (e.g., Moreno and Mayer, 2002). Overall, according to CTML, redundancy has varying effects on learning. According to the findings on verbal redundancy, adding written text to narration supports learning, but this effect depends on learners’ prior knowledge and the degree of information overlap (Adesope and Nesbit, 2012). In contrast, adding written text to narration and visualization leads to a negative redundancy effect when the written text duplicates words that are already present in the narration (Mayer et al., 2001).

### 2.1. Research questions

Based on the theoretical assumptions, we can provide a theoretical rationale for the two redundancy types. To investigate the implementation of content and working memory channel redundancy, we must consider the perspectives of instructional and cognitive psychology. From an instructional psychology perspective, we need to analyze different implementations of redundant presentation formats (e.g., image and written text, or narration and written text) and the content among these sources (e.g., duplicated or unnecessary information). From a cognitive psychology perspective, we must consider various learning and processing activities in working memory channels related to the different presentation formats.

Furthermore, it is necessary to consider interactions with existing multimedia effects. Various studies have found that level of expertise is one of the main factors moderating the redundancy effect (e.g., Lee et al., 2006). For example, information essential for learners with low prior knowledge may be ineffective (or even harmful) for knowledgeable learners (e.g., Kalyuga et al., 2000). This is called the *expertise reversal effect* (Kalyuga, 2014). According to our classification, the information might be content redundant to expert learners. Another factor is explained by the *signaling effect* (Van Gog, 2014), which refers to situations in which cues guide learners’ attention to relevant information. For example, Adesope and Nesbit (2012) showed that the implementation of narrations accompanied by short written keywords fosters learning, despite combining content and working memory channel redundancy. In contrast, narrations accompanied by identical written text impaired learning. These findings support the assumption that multimedia instructions do not have to reduce demands on cognitive processing as much as possible but should instead provide a level of processing that is neither too low (boring) nor too high (cognitive overload). In the case of Adesope and Nesbit (2012), results indicate by repeating certain information, learners can better identify what is essential to the task, which promotes efficient processing.

Based on these considerations, this review focuses on the following research questions:

1. How do studies implement the two types of redundancy?
2. Do both types of redundancy hamper learning?
3. How do the types of redundancy interact with other effects in multimedia learning?

## 3. Method

### 3.1. Literature search and coding procedures

We searched for empirical research published between January 1, 1991, and January 1, 2021. We consulted the following databases: Web of Science, Google Scholar, ERIC, ScienceDirect and PsycInfo. We used the search terms *redundancy effect, redundancy principle, redundancy, redundancy effect in multimedia learning,* and *multimedia instruction* for all databases in the fields of title, abstract, keywords, and full text (see Table 1).

**Table 1:** Keywords literature search.

Database	Search terms (number of hits)
Web of Science	Redundancy effect (2,163); redundancy principle (386); redundancy (12,908); redundancy effect in multimedia learning (81); multimedia instruction (2,830)
Google Scholar	Redundancy effect (610,000); redundancy principle (336,000); redundancy (1,150,000); redundancy effect in multimedia learning (20,600); multimedia instruction (18,100)
ERIC	Redundancy effect (208); redundancy principle (72); redundancy (1,013); redundancy effect in multimedia learning (17,944); multimedia instruction (8,523)
ScienceDirect	Redundancy effect (99,642); redundancy principle (34,926); redundancy (150,420); redundancy effect in multimedia learning (1,441); multimedia instruction (15,505)
PsycInfo	Redundancy effect (331); redundancy principle (56); redundancy (3,755); redundancy effect in multimedia learning (9); multimedia instruction (336)

**Figure 1:**
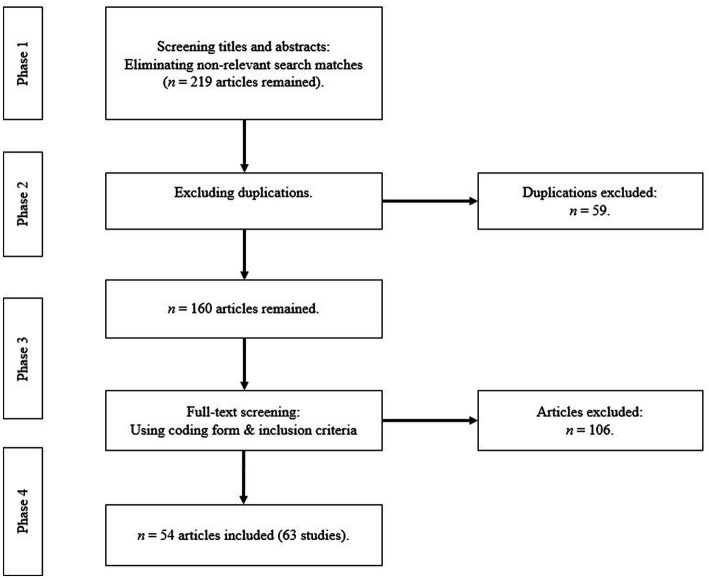
Selection process.

After the initial database searches, focused searches were conducted (see Figure 1). In the first search phase, we used the following inclusion criteria: studies that had a (quasi-) experimental design, explained their research methods, focused on the research field of multimedia learning, and were written in German or English. A screening of titles and abstracts revealed that most hits were not relevant, as many articles did not investigate the redundancy effect in multimedia learning. After eliminating irrelevant search matches, 219 articles that met all preliminary criteria remained. The next search steps (phases 2–4) included more detailed inclusion criteria to increase the comparability of the studies: studies were selected that mentioned a theoretical approach for redundancy, contained a redundant treatment and non-redundant control condition (or compared different levels of redundancy), assigned participants randomly to groups, and reported a post-test (first test after intervention). After a full-text reading, 54 articles presenting 63 studies were included in this review. Studies were excluded if they involved no empirical study, conducted a meta-analysis or review, lacked a treatment group, lacked a full set of data, involved no theoretical approach for redundancy, or were conducted in a different research field than multimedia learning (e.g., computer science or medical studies). To analyze the implementation of the two redundancy types in the studies, we developed a coding form (see Supplementary Tables S1–S2). To conduct an interrater agreement, 14 (22.2%) of the studies coded by the first coder were randomly selected to be coded by an independent coder. Per cent agreements ranged between 86 and 100% (an overview of per cent agreements is presented in the Supplementary Table S3).

## 4. Results

### 4.1. Implementation of the two redundancy types

This section illustrates the implementation of various redundant scenarios and moderating factors. To analyze the implementation of content redundancy, we focus on the provided content in the learning materials. It should be noted that it was not possible to analyze the learning materials used by the studies directly. In many studies, no information about the learning materials was available. Therefore, we relied on the authors’ statements to analyze whether some information in the materials was duplicated or unnecessary (see Supplementary Table S1). We coded the studies according to the following categories: 1 = content redundancy between visualization and textual information; 2 = content redundancy between narration and written text; 3 = no content redundancy between visualization and textual information; 4 = no content redundancy between narration and written text; and 5 = content redundancy is not mentioned.

To investigate the implementation of working memory channel redundancy, we analyzed the treatment conditions of the studies. We were able to identify four different redundant scenarios (see Supplementary Table S2) and coded the studies using the following categories: 1 = adding narration to visualizations (no working memory channel redundancy); 2 = adding written text to visualizations (working memory channel redundancy regarding visualizations and written text); 3 = adding written text to narration (working memory channel redundancy regarding written text and narration); and 4 = adding written text to narrated visualizations (working memory channel redundancy regarding visualization, narration, and written text). Table 2 illustrates the distribution of studies according to these redundant scenarios.

**Table 2:** Implementation of four redundant scenarios.

Redundant scenarios	Studies	Number of studies
Scenario 1: Adding narration to visualizations	Leslie et al., 2012, [exp. 1 and 2]; Kalyuga et al., 2000, [exp. 1 and 2].	4
Scenario 2: Adding written text to visualizations	Bobis et al., 1993, [exp.1 and 2]; Chandler and Sweller, 1991, [exp. 4 and 5]; Gellevij et al., 2002; McCrudden et al., 2014; Rop et al., 2018a, [exp. 2]; Schüler et al., 2019; Torcasio and Sweller, 2010, [exp. 1–3].	11
Scenario 3: Adding written text to narration	Bernal, 2014; Chang et al., 2011; Diao and Sweller, 2007; Dowell and Shmueli, 2008; Fenesi et al., 2015; Kalyuga et al., 2004, [exp. 3]; Liu et al., 2015.	7
Scenario 4: Adding written text to narrated visualizations	Adegoke, 2017; Aldalalah and Fong, 2010; Ari et al., 2014; Austin, 2009; Bohec and Jamet, 2008, [exp. 1]; Chu, 2006; Craig et al., 2002, [exp. 2]; Craig et al., 2004, [exp. 1]; Debuse et al., 2009; DeLeeuw and Mayer, 2008, [exp. 1 and 2]; Dooley, 2015; Dousay, 2016; Farías et al., 2014; Fenesi et al., 2015; Jadin et al., 2009; Jamet and Bohec, 2007; Kalyuga et al., 1999, [exp. 1]; Kalyuga et al., 2000, [exp. 1]; Khan and Masood, 2012; Leahy et al., 2003; Mayer and Johnson, 2008, [exp. 1 and 2]; Mayer et al., 2001, [exp. 1 and 2]; Moreno and Mayer, 2002, [exp. 2]; Munassar et al., 2010; Ozdemir et al., 2016; Pastore, 2012; Pastore et al., 2018; Rias and Zaman, 2010, Rias et al., 2014; Rop et al., 2018b, [exp. 2]; Roscoe et al., 2015; Samur, 2012; Schüler et al., 2013, [exp. 2]; Smith and Ayres, 2016, [exp. 2]; Sombatteera and Kalyuga, 2012; Stiller, 2009; Toh et al., 2010; Wallon, 2014; Wu, 2013; Yue et al., 2013, [exp. 1 and 2].	44

Some studies fulfil several scenarios. This table is based on *n* = 63 studies.

#### 4.1.1. Scenario 1: adding narration to visualizations

Scenario one illustrates four studies (6.4%) implementing content redundancy between narration and visualizations (see Table 3). In this case, both sources provide the same duplicated information. This scenario does not include working memory channel redundancy because both sources are processed in different working memory channels.

**Table 3:** Adding narration to visualizations.

Study	Content redundancy	Considered implementations (working memory channels)	Findings
Leslie et al. (2012), [exp. 1]	Between visualization and narration	Animation and narration vs. narration	Participants (with prior knowledge) in the narration only condition outperformed participants (with prior knowledge) in the animation and narration condition on transfer tasks.
Leslie et al. (2012), [exp. 2]	Between visualization and narration	Animation and narration vs. narration	Participants with prior knowledge suffered from the dual-mode presentation in contrast to novice learners.
Kalyuga et al. (2000), [exp. 1]	Between visualization and narration	Diagram and narration vs. diagram	The combination of diagram and narration was superior for novice learners but harmful for more experienced learners.
Kalyuga et al. (2000), [exp. 2]	Between visualization and narration	Diagram and narration vs. diagram	The combination of diagram and narration was harmful for more experienced learners.

Leslie et al. (2012) used learning materials on magnetism and light and compared the learning performance of younger participants (no prior knowledge) and older participants (medium prior knowledge) in two experiments. The participants received either animation and narration or narration only. The authors mentioned that the animation and narration provided the same information. Therefore, these sources were understandable in isolation (content redundancy). After differentiating their sample by age, the results indicated that older participants in the narration only condition outperformed older participants in the animation and narration condition on transfer tasks. In contrast, the differences among younger participants were not significant.

Kalyuga et al. (2000) [exp. 1] compared different instructional formats using inexperienced learner. Results indicated that learning from a fusion diagram accompanied by narration (content redundancy) was superior to a diagram only. This finding illustrated an advantage of dual-mode presentation techniques. However, after additional training, results were reversed, indicating that the advantage of the diagram plus narration disappeared, and the effectiveness of the diagram only increased on a performance test. These results were confirmed by Kalyuga et al. (2000) [exp. 2], showing that the combination of diagram and narration was essential for novice learners but harmful for more experienced learners.

#### 4.1.2. Moderating factors

Since only four studies investigate this scenario, moderating factors are limited. These studies indicate that learners’ prior knowledge moderates the effect of content redundancy (Leslie et al., 2012). All studies explained their results in light of the expertise reversal effect, which states that “information beneficial for novice learners became redundant for more knowledgeable learners” (Kalyuga, 2014, p. 577). The negative effect of content redundancy was found in participants with prior knowledge because the duplicated information became redundant and interfered with learning. Positive effects of visualization and narration were attributed to the expansion of limited working memory capacities by dual-mode presentations.

Overall, content redundancy hinders learning if participants have high prior knowledge. Therefore, it can be concluded that the positive effect of dual-mode presentations disappears due to content redundancy. In contrast, it might be possible that novice learners benefit from content redundancy or, in the case of Leslie et al. (2012), that content redundancy does not have negative effects.

#### 4.1.3. Scenario 2: adding written text to visualizations

Scenario two addresses 11 studies (17.5%) that implemented content redundancy and working memory channel redundancy (see Table 4). In this case, content redundancy exists because visualization and written text duplicate information or one source provides unnecessary information (unrelated to the learning content). Working memory channel redundancy exists because the visual channel can become overloaded by the need to scan between visualizations and written text.

**Table 4:** Adding written text to visualizations.

Study	Content redundancy	Considered implementations (working memory channels)	Findings
Bobis et al. (1993), [exp. 1]	Between visualization and written text	Diagram and written text vs. diagram	The diagram-only group outperformed the diagram and written text group.
Bobis et al. (1993), [exp. 2]	Between visualization and written text	Diagram and written text vs. diagram Diagram and written text vs. written text	The diagram-only group was superior to the diagram and written text group. The contrast between diagram and written text to written text only was not significant.
Chandler and Sweller (1991), [exp. 4]	Between visualization and written text	Diagram and written text vs. diagram	The diagram-only group outperformed the diagram and written text group.
Chandler and Sweller (1991), [exp. 5]	Between visualization and written text	Diagram and written text vs. diagram	The diagram-only group outperformed the diagram and written text group.
Gellevij et al. (2002)	Between visualization and written text	Screen capture and written text vs. written text	The screen capture and written text group outperformed the written text group.
McCrudden et al. (2014)	Between visualization and written text	Pictures and written text vs. pictures	The pictures and written text group outperformed the pictures only group on memory test. Differences on transfer test were not significant.
Rop et al. (2018a), [exp. 2]	Between visualization and written text	Image and written text vs. Image and irrelevant written text	Eye-movement data revealed that participants seemed to ignore irrelevant information with increasing task experience.
Schüler et al. (2019)	Between visualization and written text	Pictures and written text vs. written text	For spatial knowledge acquisition the combination of pictures and written text was helpful. This combination had no effect for non-spatial knowledge acquisition.
Torcasio and Sweller (2010), [exp. 1]	Between visualization and written text	Informative illustrations and written text vs. written text	The written text only group outperformed the illustrations and written text group.
Torcasio and Sweller (2010), [exp. 2]	Between visualization and written text	Informative illustrations and written text vs. uninformative illustrations and written text	Adding uninformative illustrations was superior to informative illustrations.
Torcasio and Sweller (2010), [exp. 3]	Between visualization and written text	Uninformative illustrations and written text vs. written text	No significant differences were found.

Some of the studies found negative effects showing that participants in a visualization only condition outperformed participants in a visualization and written text condition (Bobis et al., 1993 [exp. 1 and 2]; Chandler and Sweller, 1991 [exp. 4 and 5]). Other studies reported negative effects showing that participants in a written text-only condition outperformed participants in a visualization and written text condition (Gellevij et al., 2002; Torcasio and Sweller, 2010; Schüler et al., 2019 [exp. 1]). These effects were attributed to the fact that redundant materials led participants to devote mental resources to processing unnecessary information.

Torcasio and Sweller (2010) investigated the reading abilities of children. In their second experiment, they confirmed that the content of information determines whether the redundancy effect occurs. The results indicated that informative images accompanied by written text interfere with learning, unlike uninformative images accompanied by written text. In experiment three, participants received either uninformative images accompanied by a written text or written text only. Results indicated no significant differences. Since the images in this study were unrelated to the written text, the authors assumed that participants may have been able to ignore them. Rop et al. (2018a) reported comparable findings for irrelevant written text, indicating that participants seemed to ignore irrelevant information.

Furthermore, Tocasio and Sweller mentioned the low complexity of the written text. They assumed that the extraneous load retrieved by the uninformative images was not high enough to overload working memory capacity. As illustrated in experiment two, they concluded that informative images relevant to the text are more likely to invoke a redundancy effect.

By contrast, Gellevij et al. (2002) compared visualizations and written text to written text-only. They showed that content redundancy combined with working memory channel redundancy can lead to positive effects. A positive effect was also found by McCrudden et al. (2014), who investigated the effect of a written text accompanied by images that either duplicated segments of the text or did not. The results indicated that including images that duplicate text segments increased achievement scores on a memory test.

#### 4.1.4. Moderating factors

Torcasio and Sweller (2010) named the content and the complexity of information as moderating factors. They claimed that if a visualization is related to textual information, a negative redundancy effect is likely. Schüler et al. (2019) confirmed the importance of duplicated content and elucidated a further moderating factor: the knowledge that must be acquired. They compared materials with pictures corresponding with a written text to those with only pictures or only written text. The written text contained spatial or visual information that was also provided in the pictures. For the post-tests, learners needed to acquire spatial and non-spatial information. Results indicated that the learning of spatial information increased if pictures and written text contained the same spatial information, but that increase was not significant compared to learners receiving only pictures. Regarding visual information, learning increased if pictures and written text contained the same visual information, but the increase compared to learners receiving pictures only was also not significant. In contrast, a combination of pictures and spatial text hindered the acquisition of visual knowledge. McCrudden et al. (2014) identified the signaling principle (see text-based cueing; Van Gog, 2014) as a further moderating factor. They explained that the inclusion of duplicated text segments guided learners’ attention to the relevant aspects of the learning material (see Jeung et al., 1997). Therefore, content redundancy combined with working memory channel redundancy may benefit learning if the written text guides learners’ attention. This finding also aligns with the spatial contiguity principle (Mayer and Fiorella, 2014), which claims that integrating written text into a visualization promotes learning.

A further moderating factor was found in a study by Bobis et al. (1993). They demonstrated the importance of the non-redundant condition. Their study illustrated a negative redundancy effect, comparing a lesson with visualization and written text to one with visualization-only. In contrast, they found that the difference between a combination of visualization and written text and written text-only was not significant.

Overall, most studies revealed that the combination of content redundancy and working memory channel redundancy regarding visualization and written text has negative effects. However, these negative effects are influenced by non-redundant control conditions, the type of duplicated content, the complexity of information, and the knowledge that needs to be acquired. Furthermore, the positive impacts of signals and spatial contiguity can overcome the negative effects. Adding unnecessary information seems not to hamper learning if it does not overload working memory capacity.

#### 4.1.5. Scenario 3: adding written text to narration

Scenario three encompasses seven studies (11.1%) that implemented content redundancy and working memory channel redundancy (see Table 5). In this case, content redundancy is given because the narration and written text contain duplicated information. Working memory channel redundancy is given because the narration competes with the written text in working memory. From a CTML perspective, these studies investigated verbal redundancy (Mayer, 2009).

**Table 5:** Adding written text to narrations.

Study	Content redundancy	Considered implementations (working memory channels)	Findings
Bernal (2014)	Between written text and narration	Narration and written text vs. narration	The narration and written text group outperformed the narration only group.
Chang et al. (2011)	Between written text and narration	Narration and written text vs. narration	The narration and written text group outperformed the narration only group.
Diao and Sweller (2007)	Between written text and narration	Narration and written text vs. written text	The written text group outperformed the narration and written text group.
Dowell and Shmueli (2008)	Between written text and narration	Narration and written text vs. written text Narration and written text vs. narration	The combination of narration and written text improved comprehension of complex information.
Fenesi et al. (2015)	Between written text and narration	Narration and written text vs. narration	The narration and written text group outperformed the narration only group.
Kalyuga (2014), [exp. 3]	Between written text and narration	Narration and written text vs. narration	The narration only group outperformed the narration and written text group.
Liu et al. (2015)	Between written text and narration	Narration and written text vs. written text Narration and written text vs. narration	The narration and written text group outperformed the other groups on a comprehension test.

Kalyuga et al. (2004) [exp. 3] found that the combination of narration and written text had negative effects compared to narration-only. Similarly, Diao and Sweller (2007) illustrated the negative effects of the combination of narration and written text compared to written text only. By contrast, subsequent studies found positive effects (Dowell and Shmueli, 2008; Chang et al., 2011; Bernal, 2014; Fenesi et al., 2015; Liu et al., 2015). For example, Bernal (2014) investigated the learning of English as a foreign language. Learners received narration and written text with the written text containing either a full text or keywords. The control groups received either narration or the full-text version of the written text. The results revealed that learners who received narration with written keywords outperformed learners receiving only narration or only written text on a text-sound association task. The difference between the group that received narration accompanied by a full text and those who received only a written text was not significant. Since the findings of these studies are not homogeneous, we seek possible moderating factors.

#### 4.1.6. Moderating factors

Besides the influence of prior knowledge on the redundancy effect a further moderating factor is the degree of content overlap between narration and written text. Kalyuga et al. (2004) [exp. 3] found that a high degree of content overlap between narration and written text impeded learning. This result aligns with the findings of the meta-analysis on the verbal redundancy effect, which shows that narrations accompanied by identical written text impaired learning (e.g., Adesope and Nesbit, 2012).

Fenesi et al. (2015) found another moderating factor in working memory capacity as it relates to age. They compared narration accompanied by an identical written text to narration-only. After splitting their sample by age (younger participants, *M*_age_ = 18.75; older participants, *M*_age_ = 72.36), they found a positive effect. The results indicated that older participants profit from the combination of content redundancy and working memory channel redundancy regarding written text and narration. In contrast, younger participants showed no significant differences between the redundant and the narration only conditions. The authors assumed that older participants have age-related reductions in working memory for which the redundant presentation may have compensated.

As Kalyuga (2012) elaborated, the degree to which learners can control the pace of the learning material might influence the effect of redundancy. Bernal (2014) varied learning material by presenting it as either learner-paced or system-paced. She found no significant difference between the redundant and non-redundant conditions but found a significant advantage for the learner-paced conditions. This finding aligns with Kalyuga et al. (2004) [exp. 3], who found that narration and identical written text had a negative effect when presented simultaneously in a system-paced format. The moderating effect of pacing will be further discussed in section 4.1.8.

Liu et al. (2015) illustrated that written text can compensate for the transient structure of narration. They revealed differences dependent on whether the narration and identical written text are compared to narration-only or to written text-only. Results indicated that narration and identical written text increased learning compared to narration-only, but when compared to written text-only, the positive effect only applied for a recall test. Chang et al. (2011) and Dowell and Shmueli (2008) confirmed that the combination of narration with identical written text was superior to narration-only. They stated that the negative effect of the transient structure of narrations may be compensated by additional written text.

Another moderating factor is the complexity of the provided information. Dowell and Shmueli’s (2008) initial analyses revealed no significant differences until they considered the complexity of the information. When provided with highly complex information, participants in the written text and narration condition outperformed the narration-only condition.

Overall, the combination of content redundancy and working memory channel redundancy regarding written text and narration seems beneficial for learning. This positive effect is mainly moderated by prior knowledge (favoring low prior knowledge). These results confirm research insights regarding verbal redundancy that find that the benefit increases when the content overlap of narration and written text is low (favoring short written texts). The combination is also helpful when learning complex information, as the written text can compensate for the transient structure of narration. These results indicate that people with a lower working memory capacity particularly benefit from this combination. Finally, the results also show that it is beneficial to give learners control of the pace of the learning material.

#### 4.1.7. Scenario 4: adding written text to narrated visualizations

Scenario four contains 44 (69.8%) studies that implemented content redundancy and working memory channel redundancy in higher complexity. Content redundancy can occur among all three sources (e.g., all sources provide identical information), between visualization and textual information, or between narration and written text only. Working memory channel redundancy can occur because working memory can become overloaded by visually scanning between visualizations and written text and comparing the incoming streams of written text and narration.

In contrast to the previous sections, studies in this scenario still offer a high level of heterogeneity. Only three of the remaining 44 studies (6.8%) report content redundancy among all sources (see Table 6): visualization, narration, and written text (Leahy et al., 2003 [exp. 2],Farías et al., 2014; Rop et al., 2018b [exp. 2]). Therefore, an extensive analysis of the effects of redundancy is limited to these studies.

**Table 6:** Duplicated information between written text and narrated visualizations.

Study	Content redundancy	Considered implementations (working memory channels)	Findings
Farías et al. (2014)	Between visualization, narration, and written text	Images, narration and written text vs. narration and written text	The image, narration and written text group outperformed the narration and written text group on retention and transfer tasks.
Leahy et al. (2003)	Between visualization, narration, and written text	Diagram, narration and written text vs. diagram and written text	The diagram and written text group outperformed the diagram, narration, and written text group.
Rop et al. (2018b), [exp. 2]	Between visualization, narration and written text	Matching image, narration, and written text vs. mismatching image, narration, and written text vs. narration and written text	The negative effect of mismatching pictures disappeared when participants gained more task experience.

Farías et al. (2014) investigated the learning of lexical items. They compared two groups each receiving narrated verbs accompanied by identical written text, with one of those groups also receiving additional images (illustrating the action of the verb). The results indicated that, regarding retention and transfer tasks, the group receiving additional images outperformed the group with only narration and written text. Like Kalyuga et al. (2003), the authors suspected that the processing of the redundant resource occurred within available working memory capacity because learning verbs induces low element interactivity. Leahy et al. (2003) investigated learning with a complex temperature line graph. They compared two groups, each receiving self-explanatory diagram accompanied by written text, with one of those groups also receiving an additional narration (identical to the written text). Results indicated that learners with the diagram and written text outperformed those with the diagram, narration, and written text. Rop et al. (2018b) investigated the effect of irrelevant images when participants learn words from an artificial language. Groups either received matching or mismatching images accompanied by narration and written text. Results indicated that the initial negative effect of mismatching images disappeared if participants gained experience with the task.

Analyses of the remaining 41 studies revealed a subset of eight studies investigating the variation of the degree of content overlap between narration and written text (see Table 7). However, these studies did not consider content redundancy between the visualization and textual information.

**Table 7:** Varying the degree of overlap between written text and narration.

Study	Content redundancy	Considered implementations (working memory channels)	Findings
Bohec and Jamet (2008), [exp. 1]	Between written text and narration	Image, narration, and totally repeated written text vs. image, narration, and partially repeated written text vs. image and narration	Comprehension in the totally repeated written text condition was impaired. Differences between the other groups were not significant.
Dooley (2015)	Between written text and narration	Animation, narration, and complementary written text vs. animation, narration, and identical written text vs. animation and narration	No significant differences in performance test.
Mayer et al. (2001), [exp. 2]	Between written text and narration	Animation, narration, and summarized written text vs. Animation, narration, and identical written text vs. animation and narration	The animation, narration, and identical written text group performed worse on retention and transfer tests. Whereas the summarized and identical written text group did not differ.
Roscoe et al. (2015)	Between written text and narration	Animation, narration, and written text (overlap 10%) vs. animation, narration, and written text (overlap 26%) vs. animation, narration, and written text (overlap 50%)	The degree of overlap between narration and written text did not influence learning.
Sombatteera and Kalyuga (2012)	Between written text and narration	Image, narration, and full equivalent written text vs. image, narration, and phrased written text vs. image, narration, and keywords	The image, narration, and phrased written text group outperformed the other groups.
Wu (2011)	Between written text and narration	Animation, narration, and identical written text vs. animation, narration, and concise written text vs. animation and narration	The degree of overlap between narration and written text did not influence learning.
Yue et al. (2013), [exp. 1]	Between written text and narration	Animation, narration, and identical written text vs. animation, narration, and abridged written text vs. animation and narration	The animation, narration, and identical written text group performed worse. The animation, narration, and abridged written text group outperformed the animation and narration group on a transfer test.
Yue et al. (2013), [exp. 2]	Between written text and narration	Animation, narration, and identical written text vs. animation, narration, and abridged written text vs. animation, narration, and near-changed written text vs. animation, narration, and far changed written text	Benefits for the abridged written text group over the identical written text group. Near-changed written text group outperformed the identical written text group.

Results of Bohec and Jamet (2008), Mayer et al. (2001), Sombatteera and Kalyuga (2012), and Yue et al. (2013) [exp. 1 and 2], indicated negative effects of adding identical written text to narrated visualizations. Furthermore, Sombatteera and Kalyuga (2012), and Yue et al. (2013) [exp. 1 and 2], found positive effects of short compared to identical written text. In contrast, Dooley (2015), Roscoe et al. (2015), and Wu (2011) found that of the degree of overlap between written text and narration accompanied by visualizations had no effect.

The results of the remaining 33 studies that only report redundancy between narration and written text show high heterogeneity (see Table 8). Some studies indicated positive effects (e.g., Mayer and Johnson, 2008; Ari et al., 2014; Adegoke, 2017 [exp. 1 and 2]; Munassar et al., 2010; Toh et al., 2010; Samur, 2012), while others indicated negative effects (e.g., Kalyuga et al., 1999 [exp. 1]; Kalyuga et al., 2000 [exp. 1]; Mayer et al., 2001 [exp. 1 and 2]; Austin, 2009 [exp. 1]; Aldalalah and Fong, 2010; Rias and Zaman, 2010; Khan and Masood, 2012), and still others showed no significant differences (e.g., Moreno and Mayer, 2002 [exp. 2]; Craig et al., 2004 [exp. 1]; Debuse et al., 2009; Jadin et al., 2009).

**Table 8:** Adding written text to narrated visualizations.

Study	Content redundancy	Considered implementations (working memory channels)	Findings
Adegoke (2017)	Between narration and written text	Animation, narration, and written text vs. animation and narration	The animation, narration, and written text group had highest post-performance.
Aldalalah and Fong (2010)	Between narration and written text	Image, narration, and written text vs. image and narration	The image and narration group outperformed the image, narration, and written text group.
Ari et al. (2014)	Between narration and written text	Image, narration, and written text vs. Image and narration	The image, narration, and written text group outperformed the image and narration group.
Austin (2009), [exp. 1]	Between narration and written text	Animation, narration, and written text vs. animation and narration	The animation and narration group outperformed the animation, narration, and written text group.
Chu (2006)	Between narration and written text	Image, narration, and written text vs. Image and narration	No differences were found between both groups.
Craig et al. (2002), [exp. 2]	Between narration and written text	Animation (and pedagogical agent), narration, and written text vs. Animation (and pedagogical agent), and written text vs. animation and narration	The animation (and pedagogical agent) and narration group outperformed the animation (and pedagogical agent) and written text group. No differences between the other groups.
Craig et al. (2004), [exp. 1]	Between narration and written text	Animation (and pedagogical agent), narration, and written text vs. animation (and pedagogical agent) and narration	The addition of narration and written text had no effect.
Debuse et al. (2009)	Between narration and written text	Animation (and pedagogical agent), narration, and written text vs. animation (and pedagogical agent), and narration	There were no differences in learning efficacy between the groups.
DeLeeuw and Mayer (2008), [exp. 1]	Between narration and written text	Animation, narration, and written text vs. animation and narration	There were no differences regarding mental effort and difficulty between the groups.
DeLeeuw and Mayer (2008), [exp. 2]	Between narration and written text	Animation, narration, and written text vs. animation and narration	The animation, narration, and written text group had longer response times and higher mental effort ratings.
Dousay (2016)	Between narration and written text	Animation, narration, and written text vs. animation and narration	There were no differences in learning achievement between the groups.
Fenesi et al. (2015)	Between narration and written text	Image, narration, and (identical) written text vs. image and narration and (complementary) written text vs. image and narration	Younger participants benefit from the addition of complementary written text. Older participants benefit from the addition of identical written text.
Jadin et al. (2009)	Between narration and written text	Animation (and pedagogical agent), narration, and written text vs. animation (and pedagogical agent) and narration	No significant differences between the groups.
Jamet and Bohec (2007)	Between narration and written text	Image, narration, and written text vs. image and narration	The inclusion of written text led to an impairment in retention and transfer test.
Kalyuga et al. (1999), [exp.1]	Between narration and written text	Image, narration, and written text vs. image and narration	The image and narration group outperformed the image, narration, and written text group.
Kalyuga et al. (2000), [exp.1]	Between narration and written text	Image, narration, and written text vs. image and narration vs. image	The image and narration group outperformed the other groups on difficulty ratings and instructional efficacy. Results for question scores indicate no significant differences.
Khan and Masood (2012)	Between narration and written text	Animation, narration, and written text vs. animation and narration	The animation and narration group outperformed the animation, narration, and written text group.
Mayer and Johnson (2008), [exp. 1]	Between narration and written text	Image, narration, and written text vs. image and narration	The image, narration, and written text group outperformed the image and narration group on a retention test but not on transfer.
Mayer and Johnson (2008), [exp. 2]	Between narration and written text	Image, narration, and written text vs. image and narration	The image, narration, and written text group outperformed the image and narration group on a retention test but not on transfer.
Mayer et al. (2001), [exp. 1]	Between narration and written text	Animation, narration, and written text vs. animation and narration	The animation and narration group outperformed the animation, narration, written text group.
Moreno and Mayer (2002), [exp.2]	Between narration and written text	Animation, narration, and written text vs. animation and narration	Results indicate no significant differences.
Munassar et al. (2010)	Between narration and written text	Image, narration, and written text vs. image and narration	The image, narration, and written text group had significantly higher performance and motivation scores.
Ozdemir et al. (2016)	Between narration and written text	Animation, narration, and written text vs. animation and narration	Results indicate no significant differences.
Pastore (2012)	Between narration and written text	Image, narration, and written text vs. Image and narration	The image and narration group outperformed the image, narration, and written text group on learning and comprehension scores.
Pastore et al. (2018)	Between narration and written text	Image, narration, and written text vs. Image and narration	Results indicate no significant differences.
Rias and Zaman (2010)	Between narration and written text	Animation, narration, and written text vs. animation and narration	The animation and narration group outperformed the animation, narration, and written text group on a recall but not on a transfer test.
Rias et al. (2014)	Between narration and written text	Animation, narration, and written text vs. animation and narration	The animation and narration group outperformed the animation, narration, and written text group on recall.
Samur (2012)	Between narration and written text	Animation, narration, and written text vs. animation and narration	The animation, narration, and written text group outperformed the animation and narration group.
Schüler et al. (2013), [exp.2]	Between narration and written text	Animation, narration, and written text vs. animation and narration	Results indicate no significant differences neither for recall nor transfer performance.
Smith and Ayres (2016), [exp.2]	between narration and written text	Image, narration, and written text vs. image and narration	The image and narration group had significant higher learning outcomes.
Stiller (2009)	Between narration and written text	Image, narration, and written text vs. image and narration	There were no significant differences in the factual knowledge and labelling.
Toh et al. (2010)	Between narration and written text	Image, narration, and written text vs. image and narration	The image, narration, and written text group outperformed the image and narration group.
Wallon (2014)	Between narration and written text	Animation, narration, and written text vs. animation and narration	The animation and narration group outperformed the animation, narration, and written text group on retention but not on transfer.

#### 4.1.8. Moderating factors

Studies that implemented content redundancy among visualization, narration, and written text identified element interactivity as a moderating factor (Leahy et al., 2003; Farías et al., 2014). The duplication of information seems beneficial when element interactivity is low. In such cases, working memory capacity is not overloaded despite the redundancy. In contrast, when element interactivity is high, the duplication of information across all sources seems detrimental to learning.

For studies that report only on content redundancy between narration and written text, we cannot assess the extent to which, for example, the visualization also contained duplicated information because most studies did not publish their learning materials in detail. However, we assume there is at least a small overlap. For example, Austin (2009) [exp. 1] mentioned content redundancy only between written text and narration. However, an illustration of the learning material reveals that there also seems to be content redundancy between the visualization and textual information. The negative effect of using visualization, narration, and written text compared to visualization and narration may be caused by the working memory overload of needing to scan between visualization and written text and compare the incoming streams of written text and narration. Combined with content redundancy, it might be possible that the positive effect of combining narration and written text disappears when a visualization is added. From this perspective, the different types of redundancy and their moderating factors interact with each other.

Further examples were found in the studies by Mayer et al. (2001) [exp. 1 and 2], who compared animation with narration accompanied by either summarized or duplicated written text in lightning formation learning materials. The results indicated negative effects on retention and transfer tests, whether the written text was short or long. This contradicts the findings in Section 4.1.6, which showed a positive effect of content redundancy combined with working memory channel redundancy (regarding narration and written text), especially when the written text was short. We assume that a possible redundancy between the animation and written text may have overshadowed the possible positive effects of written text and narration. The findings of Moreno and Mayer (2002) [exp. 2] support this assumption. They compared animation, narration, and written text to animation and narration. Initial analyses revealed no significant differences among the conditions. However, they illustrated that content redundancy combined with working memory channel redundancy (regarding narration and written text) helped learners when the presentation was sequential and the animation was not presented simultaneously with the textual information.

Kalyuga et al. (1999) [exp. 1] and Kalyuga et al. (2000) [exp. 1] deliberately excluded content redundancy between visualizations and textual information. Results indicated that, regarding learning and cognitive load, participants learning from visualization and narration outperformed participants learning from visualization, narration, and written text. Since the textual information and visualization were both essential for understanding, the authors assumed that the negative effect was caused by split attention.

Debuse et al. (2009) and Jadin et al. (2009) included pedagogical agents as visualizations. These studies showed insignificant differences between learning materials with visualization, narration, and written text and those with visualization and narration. We suggest the following explanations for these findings. One possibility is that the positive effect of the combination of narration and written text dissipates with the addition of the pedagogical agent. Another possible explanation is that the learning materials need to be complex to cause an overload of working memory capacity. A third explanation derives from the type of visualization. If the visualization contains information unnecessary for learning (e.g., pictures of a person), it may be that learners can ignore this information. Rop et al. (2018a) [exp. 2] confirmed learners’ ability to ignore unnecessary information in learning materials. They indicated that the negative effect of narration and written text accompanied by mismatching pictures disappeared when participants were familiar with the tasks. On the other hand, inexperienced participants could not ignore unnecessary information, and their learning decreased. Therefore, the ability to ignore unnecessary information is moderated by learners’ prior knowledge.

Overall, Scenario 4 illustrates the high complexity of redundant multimedia learning environments indicating all studies combined visualization, narration, and written text; however, the implementations and results differed. Studies differed in the correspondence between visualizations and textual information, control conditions, length of text, and type of visualization (e.g., animation, images, pedagogical agents). Due to a lack of information on the learning materials, we could not provide a differentiated assignment of the various types of redundancy. Therefore, we recommend that future studies provide more detailed information on their learning materials and report how content redundancy was realized among all sources. Strictly speaking, each type of redundancy is an independent variable, and several are (unconsciously) varied in the existing studies. For example, without assessing the extent of content redundancy, we cannot analyze whether the effects of redundancy are due to unconsidered types of redundancy or other moderating effects. Nevertheless, we assume that the moderating factors presented in Sections 4.1.2, 4.1.4, and 4.1.6 also affect the studies in this scenario.

## 5. Summary, discussion, and recommendations

This review extends current insights into the redundancy effect by presenting an alternative classification that describes and characterizes different implementations of redundancy in multimedia learning environments. Overall, this review illustrates the need for a refinement of the term redundancy, as other authors have previously demanded (e.g., Baldwin, 1968; Bohec and Jamet, 2008; Dooley, 2015; Schroeder and Cenkci, 2018). This review began by distinguishing between the different theoretical assumptions of CLT and CTML regarding the redundancy effect in multimedia learning. CLT describes redundancy in a “broader “sense and relates to the logical relations of the learning content; redundancy harms learning if identical information is provided in multiple forms, if different pieces of information do not relate to each other, or if the information is unrelated to the learning content. For CTML, the redundancy effect occurs when information is presented via visualization, narration, and written text (Moreno and Mayer, 2002). In such cases, redundancy harms learning because “(a) the visual channel can become overloaded by having to visually scan between pictures and on-screen text, and (b) because learners expend mental effort in trying to compare the incoming streams of printed and spoken text” (Mayer, 2009, p. 118). Concerning these theoretical assumptions, we classify two types of redundancy: (1) content redundancy and (2) working memory channel redundancy. To investigate the implementation of these redundancy types, we reviewed empirical research on the redundancy effect of 63 studies.

In the following, the results are summarized and discussed in light of our research questions.

### 5.1. Research question 1: how do studies implement the two types of redundancy?

Studies implemented content redundancy by providing duplicated or unnecessary information among different sources (e.g., animation and narration). According to working memory channel redundancy, studies illustrated two ways of implementations: working memory channel redundancy regarding visualizations and written text and working memory channel redundancy regarding narration and written text. Overall, the implementation of both redundancy types was realized via four different scenarios: (1) adding narration to visualizations, (2) adding written text to visualizations, (3) adding written text to narration, and (4) adding written text to narrated visualizations.

### 5.2. Research question 2: do both types of redundancy hamper learning?

The implementation of content redundancy (identical information in the visualization and narration) indicates that content redundancy on its own hinders learning when participants have high prior knowledge. Novice learners, by contrast, may benefit from content redundancy, or at least that it appears content redundancy does not have negative effects. We recommend that instructions contain content redundancy when intended for novice learners, and the material is highly complex. If learners are struggling to understand complex novel information, providing content redundancy can help them to integrate the new information and reduces the cognitive effort for schema construction (e.g., an animation showing the different parts of a plant and their functions should be narrated if learners have low prior knowledge or have visual impairments). For low-complexity material, presenting the information once clearly and concisely may be sufficient for novice learners. Moreover, we recommend removing content redundancy (e.g., exclude narration from an animation) in low-complexity learning materials for learners with prior knowledge.

The analyses of the four scenarios reveal that studies implemented different types of redundancy simultaneously. Most studies that implement content redundancy combined with working memory channel redundancy (regarding visualizations and written text) reveal negative effects. However, these negative effects are influenced by the type of non-redundant condition, the type of duplicated content, and the knowledge that needs to be acquired. Furthermore, the positive aspects of signals can overcome the negative effects of this combination. For example, if learners receive an animation showing the process of photosynthesis accompanied by written text, we recommend the following: for learners with prior knowledge, include keywords if the animation is highly complex. In contrast, for low complexity, we recommend excluding the keywords. When learners have no prior knowledge, we recommend including short content-redundant written text that summarizes each step of the process if the animation is highly complex. When the animation has low complexity, the integration of keywords may be sufficient for novices. We further suggest integrating the written text into the visualization. However, Bobis et al. (1993) demonstrated that an integrated format is only beneficial if both sources are essential for learning. If the learning material has a low complexity but nevertheless contains a high degree of contentual overlap (not essential) the positive effect disappears.

Content redundancy combined with working memory channel redundancy (regarding narration and written text) seems beneficial for learning. Results align with findings on the verbal redundancy effect (Mayer, 2009). They are mainly moderated by text length (favoring short written texts), the complexity of information (favoring higher complexity), and the compensating function of written text (favoring the opportunity to re-read information missed in the narration). If complex knowledge needs to be acquired (particularly if learners struggle with reading or have visual impairments), we recommend removing redundancy regarding narration and written text when a text is lengthy or adding keywords instead of long written text to the narration. We also recommend giving learners control over the pace of the learning material. Furthermore, results indicate that learners with a lower working memory capacity particularly benefit from this combination.

Most studies implemented a combination of visualization, narration, and written text. Results indicate inconsistent results and interactions between the redundancy types. For content redundancy across all sources, results indicate that the combination of visualization, narration, and written text was helpful when dealing with complex learning material. When studies considered only content redundancy regarding narration and written text and excluded content redundancy regarding visualization and textual information, the results illustrate that learning is impaired when a visualization contains essential (not duplicated) information. In this case, we recommend excluding written text to avoid split attention. When the visualization contains unnecessary information (not related to the learning content), we recommend excluding the visualization, especially for learners with low levels of prior knowledge because they are unable to ignore irrelevant information in learning materials. In addition, the negative effect of redundancy regarding visualization and written text overshadows the positive effects of content redundancy regarding narration and written text. We recommend including visualizations, narrations, and written texts only when they are essential for understanding and when the visualization is highly complex. We further recommend implementing a short written text when the learning material is highly complex, and learners have low levels of prior knowledge. We also suggest integrating the short written text into the visualization. Otherwise, we recommend removing either the visualization or the written text. Overall, we recommend that future studies provide more detailed information on how content redundancy was implemented across different sources.

### 5.3. Research question 3: how do the types of redundancy interact with other effects in multimedia learning?

In line with Kalyuga (2012), our results indicate that the expertise reversal effect (Kalyuga, 2014) moderates the negative effect of content redundancy. It seems that novice learners benefit from redundant information. In contrast, content redundancy impedes learning for learners with high levels of prior knowledge (e.g., Leslie et al., 2012). Furthermore, the positive effects of content redundancy seem more likely when presenting information in different sensory modalities (e.g., animation and narration) what is moderated by the modality effect (Mayer, 2009). The negative effects of content redundancy combined with working memory channel redundancy (regarding visualization and written text) are moderated by either the split-attention effect (Ayres and Sweller, 2014) or the seductive details effect (Harp and Mayer, 1998). The positive effects seem to be moderated by the multimedia effect (Mayer, 2014), signaling effect (Van Gog, 2014), and spatial contiguity effect (Mayer and Fiorella, 2014). The signaling effect (Van Gog, 2014) also moderates the positive effects of content redundancy combined with working memory channel redundancy (regarding narration and written text). The verbal redundancy effect (Mayer, 2009), transient information effect (Jiang and Sweller, 2021), and expertise reversal effect (Kalyuga, 2014) moderate the negative effects of this combination.

## 6. Directions for further research

Although this review addressed some gaps in redundancy research, new questions arise. One question concerns the interactions between the different types of redundancy. Since many studies have not considered content redundancy in visual and textual information, the question remains whether working memory channel redundancy combined with content redundancy strengthens or weakens the negative effects. Another question arises regarding the quantity of content overlap. As Roscoe et al. (2015) show, the degree of content overlap between narration and written text varies significantly. Determining the overlap between visualization and textual information is much more difficult. For example, a written text may provide additional information not visible in the visualization, in which case there would be no content redundancy. However, often a text contains both identical and additional information. Future studies should pay attention to this issue and report which information is presented in the text and the visualization. One possibility for such an investigation would be to analyze which information tested in the post-test was duplicated. A first study by Albers et al. (2023) addressed this issue, illustrating that content redundancy (providing identical content) increases learning and decreases cognitive load, whereas content redundancy combined with working memory channel redundancy impedes learning and increases cognitive load. However, further research is required. Another question that arises is whether there is an influence on which information is presented in which format. For example, Hegarty (2014) has already shown that dynamic information can be better processed when presented via visualization. On the other hand, verbal information serves better when linear structures (e.g., sequences of events) must to be learned. These findings are also relevant for redundancy research. Imagine having the same information in both visualization and written text. In this case, the complexity of the information is moderated by the presentation format: if a visualization has low complexity while a written text is highly complex, we will likely see different redundancy effects depending on the control condition. If the visualization is the control condition, we will probably see a negative effect of redundancy because the visualization was already essential for understanding, and the addition of complex written text hampers learning. By contrast, if the written text is the control condition, we will likely see a positive effect because the learner will benefit from the addition of the visualization. This example shows that aspects of element interactivity must be considered. Element interactivity describes “the degree of interconnectedness between essential elements of information that should be considered in working memory at the same time” (Kalyuga, 2011, p. 2). If elements can be processed in isolation—if they do not interact—element interactivity is low. On the other hand, if elements cannot be processed in isolation—if they interact with each other—element interactivity is high (Sweller, 2010). Our review has shown that complexity (element interactivity) has a moderating effect on all types of redundancy. Future studies should examine the redundancy types separately, starting with low element interactivity, and observe how the effects change when element interactivity increases. As indicated by Sweller (2010), element interactivity reflects intrinsic cognitive load for novice learners. In contrast, for expert learners, the interacting elements are unnecessary and induce extraneous cognitive load. As suggested by Kalyuga (2014), learning material should be adapted to different levels of expertise. Therefore, future studies could provide a self-paced setting that includes the option of fading out different sources of information.

## 7. Limitations

One limitation of this review is that it includes only studies that explicitly investigated the redundancy effect and mentioned a theoretical approach to redundancy. Further analyses should investigate study designs to identify what types of redundancy are investigated instead of focusing only on the theoretical background. For example, Fenesi et al. (2016) investigated the split-attention effect and the coherence effect. They compared a complementary presentation (narration with relevant visualizations) with a split-attention presentation (narration with relevant visualizations and identical on-screen text). Based on our classification, this is an investigation of the redundancy effect. Other examples include Harskamp et al. (2007), Herrlinger et al. (2017), and Stebner et al. (2017), and even studies that investigate the seductive details effect (e.g., Lehmann and Seufert, 2017).

A further limitation is that this review provides only a first impression of the methodological similarities and differences in this area, while other important aspects—for instance, individual differences among learners (e.g., intelligence, spatial ability), different post-tests formats (e.g., retention, transfer, cognitive load, motivation), virtual reality learning environments (Baceviciute et al., 2022)—were not considered.

Another limitation is that the included studies vary greatly in how much detail they provided about the design of their learning materials. For instance, we noticed that many studies did not give specifics about the implementation of content redundancy between visualization and textual information; we were able to distinguish only whether content redundancy was implemented.

## 8. Conclusion

This review provides new contributions that have emerged in response to the research questions: It combines different theoretical assumptions of CLT and CTML and their implementation in the studies. In addition, it indicates that the empirical investigation of redundancy is sufficiently different among the studies (e.g., various combinations of modalities, different methods for presenting information, and different degrees of contentual overlap). On the one hand, this review enables scientists to classify their research on the redundancy effect and provides conceptual guidance on how to consider redundancy when designing a study. On the other hand, it illustrates educators’ options to consider redundancy during the development of learning material (not only digital but conventional lectures). Theoretically, it offers a way to define redundancy more precisely using a differentiation between two redundancy types: content redundancy and working memory channel redundancy. Empirically this review illustrates that redundancy (especially content redundancy) can support learning when used appropriately in the learning process. However, the benefits must depend on how it is applied. Too much redundancy (e.g., narration and identical written text) or redundancy presented in an unhelpful way increases extraneous cognitive load and impedes learning. Moreover, this review provides guidance to decide which types of redundancy to use or avoid, e.g., based on the complexity of the learning material, the learners’ prior knowledge or interacting multimedia effects. Moreover, it addresses practical problems in this research area (e.g., no specifics about implementing content redundancy between visualization and textual information and a lack of information on the learning materials). Finally, the review demonstrated perspectives for future studies (e.g., comparing various types of redundancy experimentally).

## Author contributions

MT and JW contributed to conception and design of the review. MT took the lead in writing the first draft of the manuscript. FS and JW provided critical feedback and helped to shape the manuscript. MT, FS, and JW contributed to the interpretation of the results, provide approval for publication of the content, and agree to be accountable for all aspects of the work in ensuring that questions related to the accuracy or integrity of any part of the work are appropriately investigated and resolved. All authors contributed to the article and approved the submitted version.

## Conflict of interest

The authors declare that the research was conducted in the absence of any commercial or financial relationships that could be construed as a potential conflict of interest.

## Publisher’s note

All claims expressed in this article are solely those of the authors and do not necessarily represent those of their affiliated organizations, or those of the publisher, the editors and the reviewers. Any product that may be evaluated in this article, or claim that may be made by its manufacturer, is not guaranteed or endorsed by the publisher.

## Footnotes

^1^Complementary redundancy: narration paired with images and written text.

^2^Partial redundancy: presentations with a low correspondence between written text and narration.

^3^Concise redundancy: shortened form of written text combined with a visualization.

^4^Note that CLT and CTML define the split-attention effect differently. CTML suggests avoiding split attention by integrating words spatially and temporally in a visualization (see spatial and temporal contiguity principle in Mayer and Fiorella, 2014). CTML further suggests distributing information across different modalities to relieve cognitive processing systems (e.g., using spoken rather than written text when adding words to a picture or animation). This effect is called the "modality effect" (Mayer, 2014). In contrast, CLT considers the logical relations among information from multiple sources. From this perspective, the modality effect only occurs under conditions where the split-attention effect occurs (Low et al., 2013)—for instance, if two pieces of information from multiple sources are unintelligible in isolation but essential for learning.
