# Supplementary material for: Two types of redundancy in multimedia learning: a literature review
Source: Front Psychol. 2023 May 4;14:1148035. doi: 10.3389/fpsyg.2023.1148035 (PMC10192876; doi:10.3389/fpsyg.2023.1148035)
Supplement: Supplementary file 1 [file Table_1.DOCX]

Supplementary Material

Two types of redundancy in multimedia learning: A literature review

Trypke, Melanie^*^, Stebner, Ferdinand, and Wirth, Joachim

*** Correspondence:** Melanie Trypke [melanie.trypke@uos.de](mailto:melanie.trypke@uos.de)

# Supplementary Tables

**Table S1**

*Coding form: Implementation of content redundancy*

| Codes (multiple entries possible) | 1= content redundancy between visualization and textual information | 2= content redundancy between narration and written text | 3= no content redundancy between visualization and textual information | 4= no content redundancy between narration and written text | 5 = content redundancy not mentioned |
| --- | --- | --- | --- | --- | --- |
| Text-based indicators (examples for possible formulations) | Written text which redescribes the diagram.  Narration describing the actions in the animation.  Written text accompanied by irrelevant images. | Spoken text which duplicates the written text.  Identical spoken and written text. | The image is not understandable without narration.  The animation is not understandable without narration.  Animation and narration are both needed for understanding. | Written text and narration provide different essential information. |  |

**Table S2**

*Coding form: Implementation of working memory channel redundancy*

| Codes (multiple entries possible) | 1= adding narration to visualizations | 2= adding written text to visualizations | 3= adding written text to narration | 4 = adding written text to narrated visualizations |
| --- | --- | --- | --- | --- |
| Type of working memory channel redundancy | No working memory channel redundancy. | Working memory channel redundancy regarding visualization and written text. | Working memory channel redundancy regarding written text and narration. | Working memory channel redundancy regarding visualization, narration, and written text. |
| Text-based indicators (examples of possible formulations) | Image compared to image and narration.  Animation compared to animation and narration. | Animation compared to animation and written text.  Image compared to image and written text. | Narration and written text compared to written text only.  Narration and written text compared to narration only. | Animation, narration and written text compared to written text only.  Image, narration and written text compared to image only. |

*Note.* It is possible that studies compare several cases (e.g., animation, narration, & written text vs. animation & narration and, at the same time, animation & written text vs. animation). In this case a multiple entry, e.g., 1 & 3, will be entered. The studies sometimes differ in terminology: for instance, ‘image’ includes static pictures, pictures, illustrations, diagrams, & PowerPoint slides; ‘animations’ includes videos; ‘narration’ includes spoken words; and ‘written text’ includes on-screen text, printed text, & verbal labels.

**Table S3**

*Interrater agreement (based on 14 studies).*

| Dimension |  | Per cent agreement |
| --- | --- | --- |
| Implementation of content redundancy | 0.86 | |
| Implementation of working memory channel redundancy | 1.00 | |
